# An Identity Recognition Model Based on RF-RFE: Utilizing Eye-Movement Data

**DOI:** 10.3390/bs13080620

**Published:** 2023-07-26

**Authors:** Xinyan Liu, Ning Ding, Jiguang Shi, Chang Sun

**Affiliations:** Public Security Behavioral Science Lab, People’s Public Security University of China, Beijing 100038, China; 2021211122@stu.ppsuc.edu.cn (X.L.); 2020211540@stu.ppsuc.edu.cn (J.S.); sunchang1994@foxmail.com (C.S.)

**Keywords:** eye-movement features, simulated crime experiment, identity recognition, random forest, recursive elimination

## Abstract

Can eyes tell the truth? Can the analysis of human eye-movement data reveal psychological activities and uncover hidden information? Lying is a prevalent phenomenon in human society, but research has shown that people’s accuracy in identifying deceptive behavior is not significantly higher than chance-level probability. In this paper, simulated crime experiments were carried out to extract the eye-movement features of 83 participants while viewing crime-related pictures using an eye tracker, and the importance of eye-movement features through interpretable machine learning was analyzed. In the experiment, the participants were independently selected into three groups: innocent group, informed group, and crime group. In the test, the eye tracker was used to extract a total of five categories of eye-movement indexes within the area of interest (AOI), including the fixation time, fixation count, pupil diameter, saccade frequency, and blink frequency, and the differences in these indexes were analyzed. Building upon interpretable learning algorithms, further investigation was conducted to assess the contribution of these metrics. As a result, the RF-RFE suspect identification model was constructed, achieving a maximum accuracy rate of 91.7%. The experimental results further support the feasibility of utilizing eye-movement features to reveal inner psychological activities.

## 1. Introduction

When a person’s eyes start to avert, you have to be careful. Maybe he/she is starting to think about how to make up lies. How to accurately identify lies has been the topic of human research for ages. The human method of identifying lies has gone through three stages [1]: the divine knowledge method, the criminal knowledge method, and the instrument knowledge method, among which the human knowledge method runs through. The divine knowledge method refers to relying on the gods to judge the truth or falsity, while the criminal knowledge method relies on physical torture or disguised physical torture to identify the truth or falsity of words. Compared with the original divine knowledge method and the cruel criminal knowledge method, the human knowledge method relies on human experience and wisdom to make judgments without the use of external means, but this inevitably leads to subjective assumptions and is difficult to promote due to certain conditions. In contrast to the human recognition method, the instrument knowledge method identifies lies by means of equipment, mainly by recording physiological changes controlled by the autonomic nervous system, which can be objectively measured and recorded with objectivity and stability, thus reducing the impact of subjective errors in judgment caused by the internal tendencies of police officers.

With the rapid development of psychology, neurology, computer science, and other disciplines, tools and methods for identifying lies have been gradually improved. In 1921, Larson developed an instrument capable of recording blood pressure and respiration by combining a sphygmomanometer and a respirometer [2]. In 1926, Leonarde Keeler further improved the polygraph by combining a GSR device with the original foundation and also enhanced the portability of the polygraph [3]. Reid further improved the accuracy of the polygraph by simultaneously recording the test participants’ blood pressure, pulse, respiration, skin current, and muscle activity in 1945. Until the 1960s, polygraphs began to develop in the direction of electronics, and in this process, more and more researchers used eye-tracking technology to conduct tests due to its stability and objectivity, low interference, and its ability to eliminate the psychological defenses of offenders to a greater extent. At the same time, on the basis of accumulated practical experience, experts developed a variety of targeted polygraph methods such as GKT (Guilty Knowledge Test), QCT (Question Crossing Test), and CQT (Control Question Test). The GKT is also employed as an experimental method in this study. Derived from the “Odd ball task” paradigm, the GKT consists of probe stimuli and irrelevant stimuli. The probe stimuli primarily contain information related to the occurrence of a crime, while the irrelevant stimuli are random and unrelated to the measurement target. The objective of the GKT is to elicit participants’ recollection of knowledge and personal experiences. If a participant lacks certain knowledge or experiences, distinct indicator features will be observed before the testing apparatus, and these indicator differences are utilized to determine the participants’ association with the case or their involvement in criminal activities.

Among them, the GKT has received extensive research support, primarily focused on simulated experiments. Ben-Shakhar and Furedy [4] summarized ten studies in which 84% of criminals and 94% of innocent individuals were correctly classified. Elaad [5] conducted a review of 15 simulated crime studies, revealing that the average detection rate for guilty participants was 80.6%, while for innocent participants, it was 95.9%. It is noteworthy that among these 11 studies, no false positive identifications were observed, meaning innocent suspects were not wrongly classified as guilty. Nevertheless, due to the limitations of the GKT itself and considering the actual operation process, this paper also encountered some difficulties in the experimental process: (1) there was a fragmentation between simulated crime scenes and real crime scenes, which was not realistic enough; (2) there were false confessions by the perpetrators, and the previous way through oral questioning and identification was not objective and accurate enough; and (3) there were many dimensions of experimental data, which were difficult to analyze.

To overcome these problems, we improved the experiment. (1) In order to create a more realistic crime environment, a “task-reward” stimulus was introduced in the experiment. Prior to the formal experiment, a questionnaire was used to investigate how much “crime gain” was needed to motivate people to commit crimes when they chose their own identity to complete the “crime task” and thus underwent different psychological pressures. (2) In the test session, we used a mixture of portraits, objects, and scenes and mixed the pictures of the crime with the accompanying pictures for more objectivity. (3) Instead of using traditional common indicators, such as skin electricity, respiration, heart rate, and blood pressure, the participants were experimented on with the less disturbed eye-movement technique, and by extracting several eye-movement indicators, the experimental data were processed with great difficulty when analyzing multi-dimensionally, and the interpretable machine learning method was adopted.

In this paper, based on eye-tracking technology, we obtain the eye-movement indicators of three groups of people who choose to play innocent, informed, and criminal identities in simulated scenario crimes, these being their eye-movement indicators while viewing portraits, objects, and scene pictures. We then use the obtained indicators to construct a suspect identification model. The second part focuses on suspect recognition, eye-tracking technology recognition, and interpretable learning applications. The third part details the experimental design and the experimental results obtained using statistical analysis methods. The fourth section then describes the random forest model based on the RFE algorithm and analyzes which features contribute more to the prediction results of the model so that more accurate identification can be achieved with fewer metrics.

## 2. Literature Review

### 2.1. Suspect Identification Research

Footprints are one of the most common pieces of real evidence at crime scenes and play an important role in determining the identity of a suspect [6]. In Western countries, police consult doctors to solve cases in the hope that they can help analyze the footprints found at the crime scene and provide direction to identify the perpetrator [7]. However, it can be a challenge for investigators because it may be incomplete or have its clarity affected by external conditions, thus making identification more difficult. Currently, the Gunn Method, the Optical Center Method, the Overlay Method, the Reel Method, etc., are commonly used for footprint analysis [8]. Norman Gunn was the first to develop footprint analysis by taking linear measurements of footprints at different locations [9]. Robbins et al. created regressions of the length and width of footprints on height and weight by collecting footprint data from 500 people as well as height and weight information, noting that each person’s footprint is unique [10]. Kennedy [11,12] et al. collected flat footprints from 3000 people from 1995 to 2005, and by selecting representative populations, collecting samples repeatedly at regular intervals, and using computers to extract a total of 38 eigenvalues for input into the footprint library with the help of detectives, a mismatch rate of up to one part per billion of the footprints was achieved using this method. With the development of information technology, some researchers began to use pressure sensors to obtain footprints. Jung et al. used pressure sensors to collect footprint samples from 120 people and extracted features such as footprint area and pressure center to identify the participants with an accuracy of 97.8% [13]. In addition, there are many tracking methods, such as face recognition, that are used in many scenarios to help track drug traffickers, find missing persons, monitor suspects, etc. Abudllah et al. used PCA to implement face recognition, especially for detecting cases where no fingerprints were left at the crime scene [14]. Kakkar and Sharma constructed a crime recognition system using the Haar cascade classifier, which tags images by finding specific Haar features in the image and allows for the comparison of scanned images with still images or video streams to complete the detection [15]. When performing identification, lip prints were confirmed to be unique to a person as well [16]. By extracting the lip prints of 100 participants, Dwivedi et al. not only confirmed that the difference between male and female lip prints was statistically significant, but also that the matching rate could reach 82% with good identification [17]. Scent can also be a vehicle to identify suspects. In the experiment of Penn et al., 197 individuals were sampled every two weeks for ten weeks, and the results showed that for a correct identification, the whole profile needs to be sampled [18]. The experiment by Cuzuel et al. was performed by sampling hand odors and characterizing them using GC × GC–MS chromatography. Further, using a Bayesian framework made it possible to provide probability estimates of odor samples coming from the same person with an accuracy higher than 98%, which is suitable for application in forensics [19].

### 2.2. Eye-Movement Technology in Identifying Criminal Suspects

As technology continues to evolve, more and more scholars are using eye-tracking technology for human identification. Papesh found that pupil diameter is positively correlated with the cognitive load on the person processing the stimulus, and that as the cognitive load increases, the diameter of our pupil increases accordingly [20]. It is also important to note that the change in pupil diameter is not controlled by subjective consciousness, which provides strong evidence that changes in pupil diameter can determine whether or not a person is lying. In their experiment, Walczyk et al. divided the participants into three groups: honest, unrehearsed lying, and rehearsed lying [21]. By watching a video of a real crime and answering questions, the study found that the honest group had the fastest response time and the smallest mean pupil diameter, indicating the least cognitive load. In a simulated crime scenario experiment, Rebecca found that the pupil diameter of perpetrators was significantly larger than that of non-perpetrators when viewing pictures related to the crime scene, while no significant difference was observed when viewing pictures unrelated to the crime scene [22]. Ryan analyzed the differences in eye-movement indicators between participants viewing familiar and unfamiliar faces and found that the fixation time on familiar faces was significantly longer than that on unfamiliar faces and the number of gaze points within the familiar face area was significantly higher than that in the unfamiliar face area [23]. By testing the saccade frequency of the same participants, Vrij et al. found that the saccade frequency was higher when telling lies than when not lying, but there was not enough evidence to prove the significance level [24].

### 2.3. Application of Machine Learning in Identity Recognition

In addition, some scholars also introduced machine learning into the field of predicting crime and identifying criminal suspects. Wang et al. classified fingerprints based on deep neural networks to classify and predict suspicious fingerprints and take softmax regression to improve classification accuracy [25]. Li extracted features based on criminal records, constructed a model using SVM, calculated the similarity between predicted features and the features of people in the library in the alternative library, and then predicted the suspects [26]. Based on the analysis of property crime patterns, Li et al. further explored the nonlinear relationship between factors and property crime using a neural network model and developed a prediction model [27]. Gruber et al. generated a graphical network based on Bayesian to describe the behavior patterns of suspicious and non-suspicious users to identify suspected criminal cell phone users, and the experimental results showed that the false positive rate was less than 1% [28]. Gao et al. used five machine learning methods to analyze terrorist attack data with a maximum prediction accuracy of 94.8%, helping to target criminals for effective combat [29]. Zemblys et al. trained the classifier based on random forests for fixation time, sweep, and other eye-movement events, and the classifier performance approximated that of manual coding by eye-movement experts and had a lower error rate compared to previous studies [30]. Zhang et al. trained the model based on the XGBoost algorithm and used Shapley to explain the contribution of the variables in it to derive a ranking of factors influencing regional crime, which helps the police take targeted measures for each location [31].

In summary, suspect identification research has focused on developing effective methods to distinguish between guilty and innocent individuals. Studies utilizing eye-movement technology have shown promising results in this regard. By analyzing eye-movement patterns during the viewing of stimuli such as portraits, objects, and scenes, researchers have been able to extract valuable indicators for identifying suspects. The application of machine learning algorithms has greatly enhanced the analysis and interpretation of eye-movement data for identity recognition. These algorithms enable the extraction of meaningful features from eye-movement patterns and the development of robust models for suspect identification. Machine learning techniques, combined with eye-movement technology, offer a powerful approach to improving the accuracy and efficiency of suspect identification processes. In conclusion, the integration of suspect identification research, eye-movement technology, and machine learning algorithms holds significant potential for enhancing identity recognition in criminal investigations. Therefore, by utilizing the GKT methodology in designing simulated crime experiments, capturing various eye-movement indicators through eye-tracking technology, and employing machine learning methods to construct a suspect identification model, we can provide support for existing research findings and enhance the accuracy of suspect identification.

## 3. Experiment

### 3.1. Experimental Design

To further investigate the effectiveness of eye movements in inferring participants’ identity, the experimental procedure was designed according to the GKT. A total of 98 participants were recruited for the experiment, including 59 males and 39 females. A total of 8 participants took part in the preliminary experiment, while 90 participants participated in the formal experiment. All participants participated voluntarily. During the formal experiment, seven participants were unable to capture their signals by the eye tracker due to fatigue and loss of positioning, so the experimental data were excluded. A total of 83 valid data points were finally collected, with an age range of 18–24 years. These participants were recruited from the sophomore, junior, and first-year graduate students at the People’s Public Security University of China. Among the collected valid data, there were 53 male and 30 female participants, all of whom reported no history of illness or visual impairments. The experiment was approved by the Academic Ethics Committee of the People’s Public Security University of China. All participants signed an informed consent form prior to their participation in the experiment. We assure you that the data collected will only be used for the purpose of experimental analysis and will not be utilized elsewhere or disclosed to any third parties.

In addition, in order to make the experimental scenario more realistic and to further enhance the tension and excitement of the perpetrators in the experiment, a “task-reward” stimulus was introduced before the formal experiment. A questionnaire was used to explore how much reward could attract the participants to complete the simulated crime tasks on their own. A total of 95 questionnaires were collected, including 47 males and 48 females, and the results showed that the participants’ choices in this case were more in line with experimental expectations: “Choose task A to complete all the processes as required to get paid 30 yuan. Choose task B, you will bear the risk of failure, if you fail you will only get 15 yuan. If you succeed, you will receive the reward amount of 60 yuan.” Task A involves finding the target files required by the opposing company, verifying the matching identification numbers, and secretly carrying the files and USB drive to Room C to complete the rendezvous task, if you choose to disclose the trade secrets. Task B involves refusing the temptation of monetary compensation and not disclosing the target files to the rendezvous personnel, if you choose to reject the salary reward.

#### 3.1.1. Experiment Preparation

According to GKT, the method of free browsing was used, and the types of stimuli included portraits, objects, and scenes. Among them, 4 images were related (1 portrait, 1 object, 2 scenes) and 22 images were unrelated (11 portraits, 5 objects, 6 scenes). This experimental method involved fixing the presentation time for each image and allowing participants to freely explore the pre-determined experimental content while wearing an eye-tracking device. The content was played in a loop from start to finish. The purpose of selecting portraits, objects, and scenes as stimuli was to explore the relationship between eye-movement patterns and identity recognition from multiple perspectives. Participants, during the simulation of a criminal scenario, may exhibit psychological traces due to their nervousness when encountering people, objects, and environments. By presenting these crime-related images, their memories can be triggered, while innocent individuals, who are unaware of the scenario, would not experience heightened emotional tension. By studying different types of stimuli, we extracted eye-movement indicators from participants’ viewing processes of these images, allowing us to obtain a more comprehensive and accurate understanding of the topic.

The instrument used for the experiment was a desktop eye-tracking device from SMI, with a set sampling rate of 120 Hz and a resolution of 1024 × 768. The experimental materials were edited in advance by Experiment Center, data were collected by iView X, and the required data were finally exported using Begaze. We utilized Begaze to open the project file and extract the required eye-movement data by defining areas of interest and performing other operations. In addition, Begaze software facilitated various visualization analyses such as generating gaze heatmaps and bar charts. For our research, after extracting the data using Begaze, we employed common descriptive statistics to calculate the mean, variance, and other relevant measures for each eye-movement indicator category.

#### 3.1.2. Experimental Procedure

**Figure 1:**
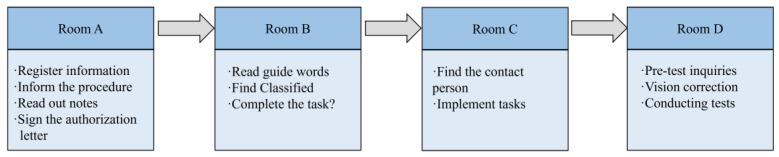
Experimental procedure.

Four rooms were set up in this experiment, and participants entered Room A, Room B, Room C, and Room D sequentially during the experiment according to the flow. In Room A, participants completed the registration process, providing relevant information and receiving instructions regarding the experiment’s procedures. Room B served as the primary setting for the simulated experiment. Participants read prompts and selected tasks with varying degrees of risk based on the provided prompts. In Room C, participants located a contact person based on the prompts and delivered the items required for the task, thereby completing the assigned task. Room D involved pre-test inquiries and eye-tracking tests. Room D was used for testing, and before the eye-movement test started, participants were calibrated to ensure that both X- and Y-axis deviations were less than 1° before proceeding. Each picture was presented for 2000 ms and then presented “+” for 500 ms, alternating in turn. The testing phase requires waiting for all the pictures to finish playing. The experimental flow is shown in Figure 1.

### 3.2. Data Analysis

At the end of the experiment, the experimental data were extracted using Begaze, and a total of 83 valid data points were obtained by combining the participants’ experimental performances and eye-movement records, including 28 valid data points for the perpetrator group, 30 valid data points for the informed group, and 25 valid data points for the innocent group. For a more intuitive representation, we defined each set of data GR.(CTPI), where TPCI all represent different meanings, specifically.

**Figure 2:**
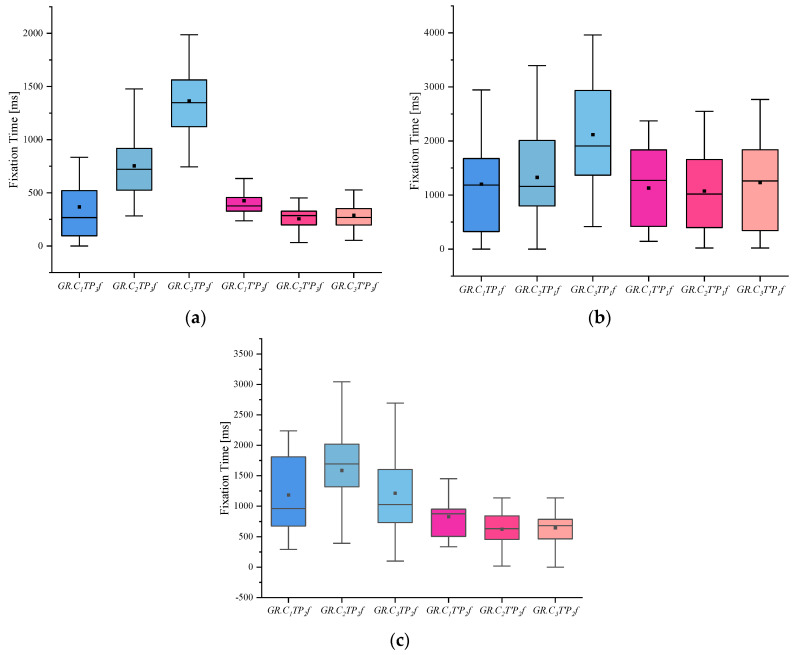
Results of Fixation Time: (**a**) Scene Fixation Time, (**b**) Portrait Fixation Time, and (**c**) Object Fixation Time.

**Figure 3:**
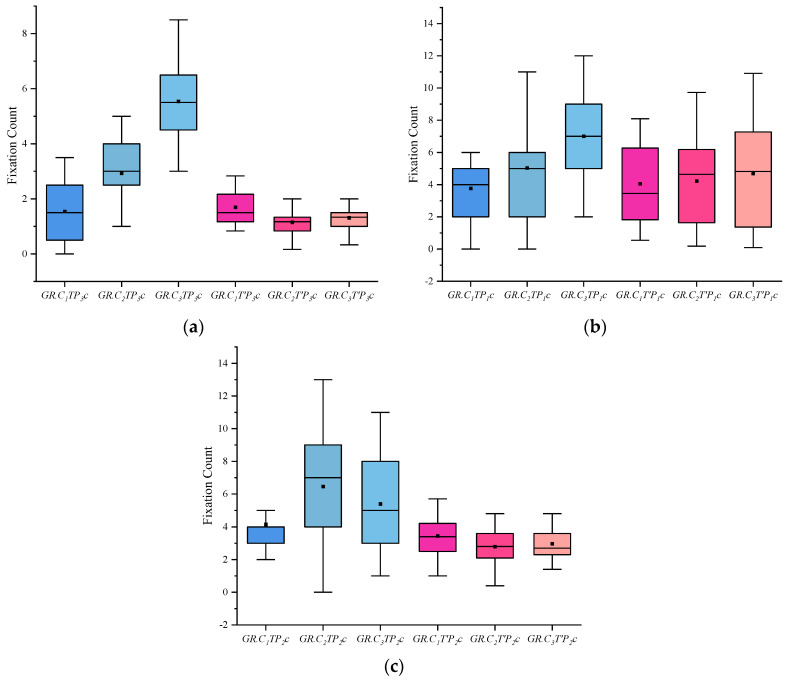
Results of Fixation Count: (**a**) Scene Fixation Count, (**b**) Portrait Fixation Count, and (**c**) Object Fixation Count.

**Figure 4:**
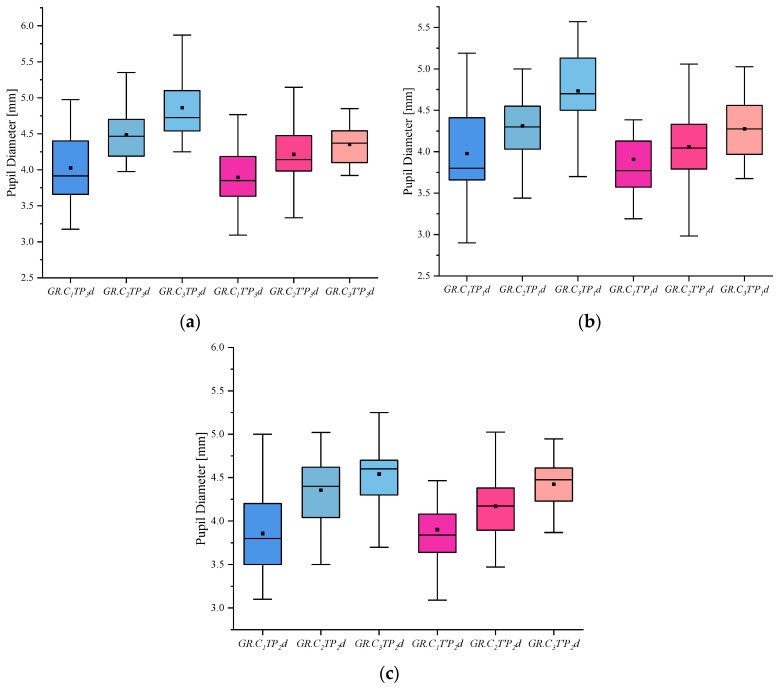
Results of Pupil Diameter: (**a**) Scene Pupil Diameter, (**b**) Portrait Pupil Diameter, and (**c**) Object Pupil Diameter.

**Figure 5:**
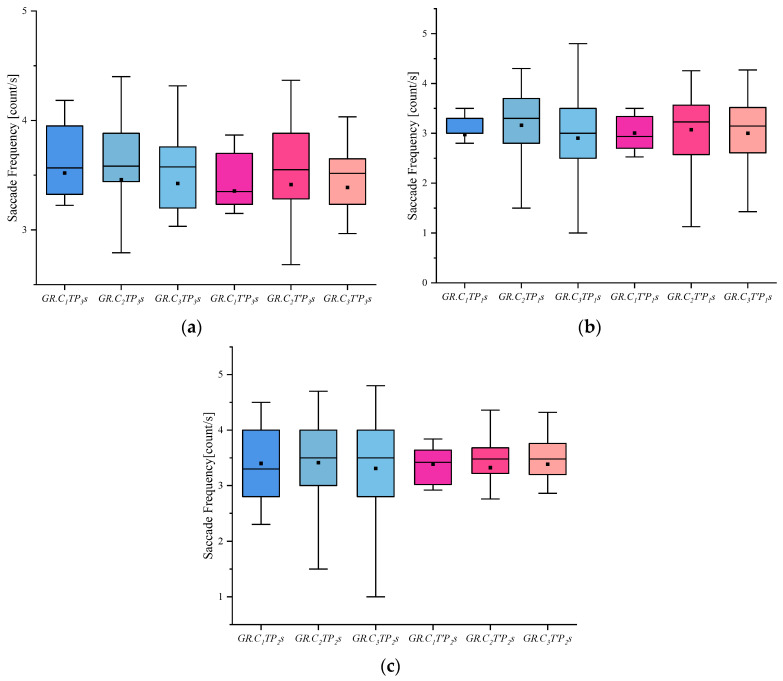
Results of Saccade Frequency: (**a**) Scene Saccade Frequency, (**b**) Portrait Saccade Frequency, and (**c**) Object Saccade Frequency.

**Figure 6:**
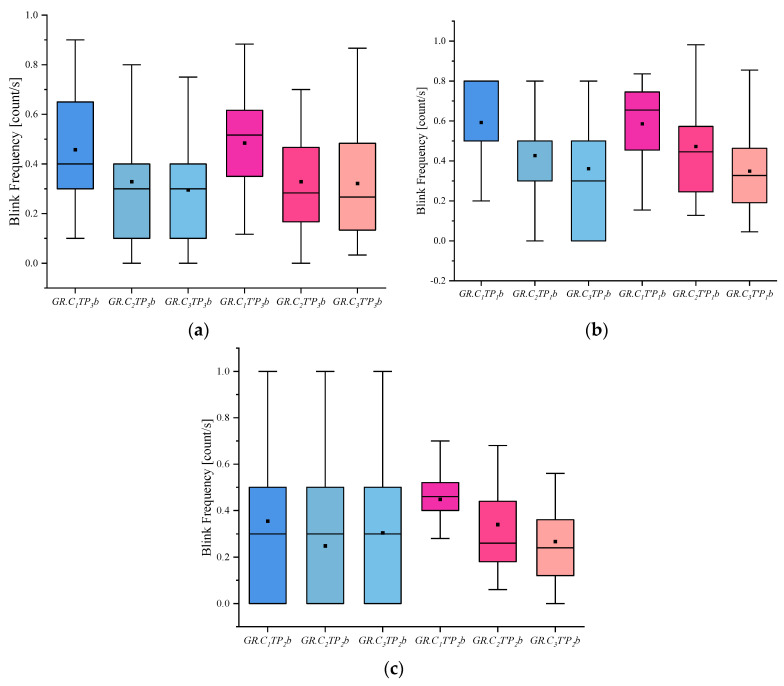
Results of Blink frequency: (**a**) Scene Blink Frequency, (**b**) Portrait Blink Frequency, and (**c**) Object Blink Frequency.

(1) C indicates the category. The innocent group is denoted by C_1, the informed group by C_2, and the crime group by C_3;

(2) T indicates whether the picture is a target picture, i.e., whether it is related to the crime. Target pictures are denoted by T, and non-target pictures are denoted by T’;

(3) P indicates the kind of picture. Portrait pictures are denoted by P_1, object pictures by P_2, and scene pictures by P_3;

(4) I indicates the eye-movement index indicator. f is used for gaze duration, c for gaze frequency, d for pupil diameter, s for eye-hopping frequency, and b for blink frequency. By delineating the area of interest (AOI) in the picture, the participants’ gaze duration, gaze frequency, pupil diameter, blink frequency, and eye beat frequency in the AOI were extracted, and the mean and standard deviation were calculated for each of the five indicators. The experimental results are shown in Figure 2, Figure 3, Figure 4, Figure 5 and Figure 6. (See Appendix A Table A1, Table A2 and Table A3 for descriptive statistics of the data.)

### Analysis of the above Indicators

(1) From the gaze duration indicators, we can see that the people in the crime group generally had more fixation time than the other two groups when viewing the case-involved pictures and showed significant differences in the case-involved and non-case-involved pictures. The informed group also showed a more obvious tendency when viewing the case-involved and non-case-involved pictures.

(2) The fixation count indicators show that when viewing the case-involved pictures, both the crime group and the informed group were higher than the innocent group, and the crime group was more obvious. When viewing the non-case-involved pictures, all three groups did not differ significantly.

(3) The pupil diameter index shows that the innocent, informed, and crime groups showed an increasing trend in both involved and uninvolved pictures, and the crime group had the largest pupil diameter when viewing the involved pictures under the same stimulus conditions.

(4) It can be seen from the blink frequency index that whether pictures and groups were involved in the case on the eye-hopping frequency had no significant difference in results.

(5) The blink frequency index shows that people in the innocent group blinked slightly more frequently than the other groups, whether they were viewing the involved or uninvolved pictures. The crime group, on the other hand, showed a slightly lower blink frequency than the informed and innocent groups. This could also confirm that there was a certain degree of blink inhibition when people lie, and thus blink frequency was lower than normal.

## 4. An RF-RFE Model Based on RFE Interpretable Machine Learning

### 4.1. Model Construction

Random forest, proposed by Breiman et al., is constructed using decision trees as the base learner. It consists of multiple decision trees and a large collection of tree-based estimates [32]. During the training process, a portion of the sample set is randomly selected with a release back, and some features are selected for training. The Booststrap training set is generated using the bagging method, and at last, simple voting is used as the basis for classification. Different sample sets are drawn for each tree, and different results are trained. The random forest model construction is roughly divided into four steps: (1) random sampling to train the decision tree; (2) randomly selecting the features of attributes to determine the attributes of node splitting; (3) repeating step 2 until the decision tree grows completely; and (4) building a large number of decision trees until the number meets the design requirements, merging the trained decision trees, and finally constructing the random forest model. The specific establishment process is shown below (see Algorithm 1).
**Algorithm 1:** Random Forest construction process$\mathrm{Input}:\;\mathrm{training}\;\mathrm{set}\;D=\{\left(x_{1},y_{1}\right)$$,\;\left(x_{2},y_{2}\right)$,…, $\left(x_{m},y_{m}\right)$};        $\mathrm{Attribute}\;\mathrm{set}\;A=\{a_{1},a_{2},\ldots ,a_{d}$}.Process: Function TreeGenerate (*D*, *A*)1: Generate node;2: **if** The samples in *D* all belong to the same category *C* **then**3:   Mark node as a class *C* leaf node; **return**4: **end if**5: **if**
*A* = ∅ **OR** The samples in *D* take the same value on *A*
**then**6:   Mark the node as a leaf node, category is marked as the class with the largest number of samples in *D*; **return**7: **end if**$8:\;\mathrm{Select}\;\mathrm{the}\;\mathrm{optimal}\;\mathrm{division}\;\mathrm{attribute}\;a_{*}$ from *A*;$9:\;\mathrm{for}\;\mathrm{Each}\;\mathrm{value}\;a_{*}^{v}$ $\;\mathrm{in}\;a_{*}\;$ **do**$10:\;\;\;\mathrm{Generate}\;a\;\mathrm{branch}\;\mathrm{for}\;\mathrm{node};\;\mathrm{let}\;D_{v}$ $\;\mathrm{denote}\;\mathrm{the}\;\mathrm{subset}\;\mathrm{of}\;\mathrm{samples}\;\mathrm{of}\;D\;\mathrm{on}\;a_{*}$ $\;\mathrm{that}\;\mathrm{take}\;\mathrm{the}\;\mathrm{value}\;a_{*}^{v}$;$11:\;\;\;\mathrm{if}\;D_{v}$ is empty **then**12:     Mark the branch node as a leaf node and its class as the class with the most samples in *D*; **return**13:  **else**$14:\;\;\;\mathrm{With}\;\mathrm{TreeGenerate}\;(D_{v}$ $,\;A\;\backslash \;\{a_{*}$}) as branch node15:  **end if**16: **end for**Output: a decision tree with node as the root node

In this paper, to better explore the effect of these features on the identity recognition model, we refer to the Recursive Feature Elimination (RFE) algorithm, which is one of the wraparound feature selection algorithms. The RFE algorithm trains all feature variables, ranks each feature’s relevance during training, and then cross-validates to determine the original feature subset’s classification accuracy. The lowest-ranked features are then deleted, and the process is repeated with a new feature set. Until the feature subset is empty, the importance rating of all features is completed. The process is shown as follows (see Algorithm 2):
**Algorithm 2:** RFE algorithm process1: **for** the results of each resampling **do**2:   Divide the data into training set, test set by resampling;3:   Train the model in the training set using feature variables;4:   Evaluate models with test sets;5:   Calculate and rank the importance of each feature variable;6:   Remove the least important features;7: **end**8: Decide on the proper number of characteristic variables9: Estimation of the set of feature variables ultimately used to build the model

When entering the variables into the suspect identification model, the previous definition of the data was combined. We took as input whether participants viewed the picture as a target, the type of picture, and the type of eye-movement indicator. The output of the model is the participant’s group type. That is, we entered GR.(TPI) into the model with a total of 30 variables (2 × 3 × 5) according to the meaning of PCI. To determine the importance ranking of the 30 indicators, the experimental 30 variables were ranked using the RF-RFE algorithm, as shown in Table 1.

### 4.2. Analysis of Results

In the training phase, the eye-movement data of all participants are used as input to construct a classification model, which is used to predict which group a participant belongs to. In which, the number of features is added sequentially according to the feature ranking obtained by the RF-RFE algorithm, and new training tests are performed by adding features successively until all 30 features are added. Combined with the accuracy performance of the previous classifier on the six classifications, in the subsequent RF-RFE operation, we mainly target the last five dichotomies. The specific results are shown in Appendix A Table A4. Several indicators were used to assess the effectiveness of the model when analyzing the model results.

**Figure 7:**
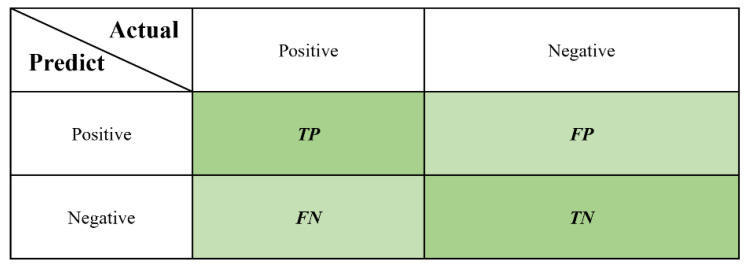
Diagram of confusion matrix [33].

(1) Accuracy (ACC): The confusion matrix can be used to compare the classification results by visualization. The predicted category and true category are represented by rows and columns, respectively, as shown in Figure 7. *TP* (*True positive*) means the prediction is true and the actual is true, *FP* (*False positive*) means the prediction is true and the actual not, *TN* (*True negative*) means the prediction is false and the actual is false, and *FN* (*False negative*) means the prediction is false and the actual is true.

The accuracy rate is expressed as the proportion of correctly predicted (whether true or false) samples to the whole sample. It is expressed in the model as the proportion of correctly predicted participant identities to all participants, and its calculation formula is shown in Equation (1).

(1) $$Accuracy=\frac{TP+TN}{TP+FP+FN+TN}$$

(2) The Kappa coefficient is a measure of classification accuracy based on the confusion matrix, which can be tested for consistency, and its calculation result interval is [−1, 1], but usually takes a value range greater than 0. And the larger the Kappa is, the better the consistency is. The Kappa calculation formula is shown in Equation (2).

(2) $$Kappa=\frac{p_{0}-p_{e}}{1-p_{e}}$$

Among them, $p_{0}$ is the ratio of the sum of the correctly classified sample size to the total, which is the value of ACC. $p_{e}$ supposes the number of actual samples in each category is *a*_1_, *a*_2_, *a*_3_, …, *a*_m_, the number of samples in each category of the prediction result is *b*_1_, *b*_2_, *b*_3_, …, *b*_m_, and the total number of samples is *n*. Then, $p_{e}=\frac{a_{1}\times b_{1}+a_{2}\times b_{2}+\ldots +a_{m}\times b_{m}}{n\times n}$.

**Figure 8:**
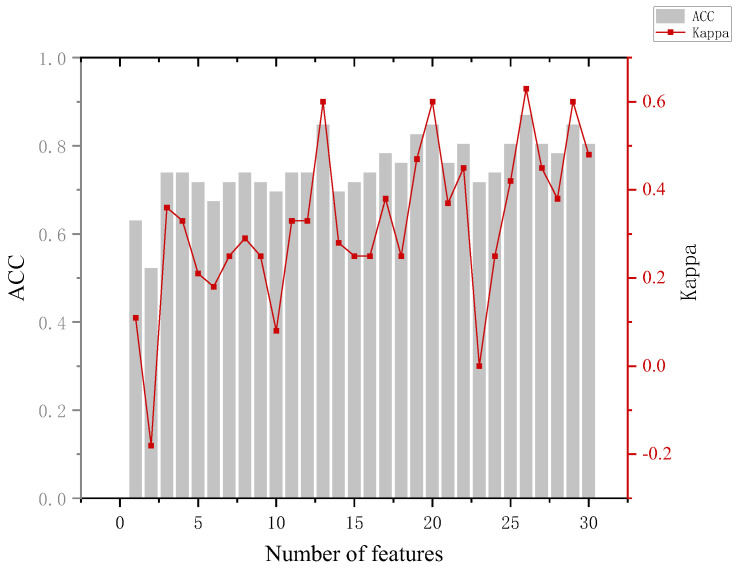
Results of the innocent and informed groups.

The analysis of Figure 8 leads to the following conclusion. When distinguishing between the innocent and informed groups, the highest model accuracy of 84.8% was achieved with 13 features and 20 features. At the same time, we can see that with the addition of the $GR.\left(TP_{1}I_{b}\right)$ feature, the accuracy of the model improved by about 11%. Back to the data, we can see that the blink frequency feature of the portrait involved in the case is significantly larger in the innocent group than in the informed group. This can corroborate the blink suppression phenomenon [34] and provide a basis for determining whether a person is lying or not.

**Figure 9:**
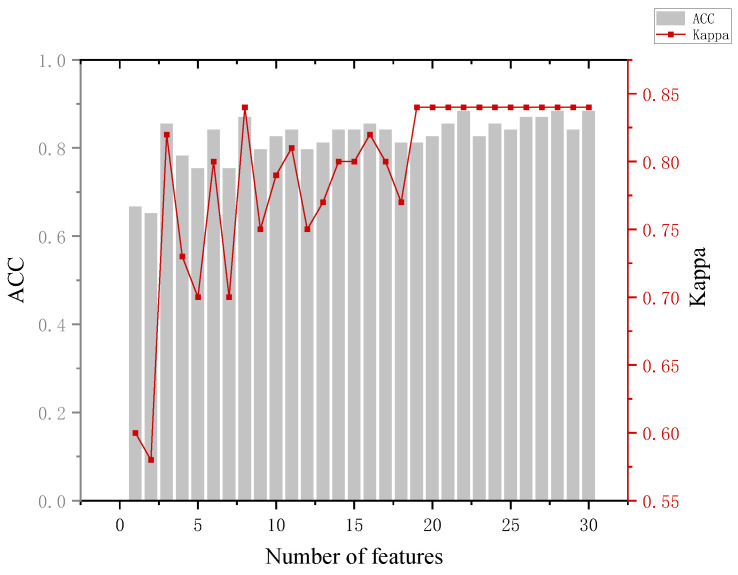
Results of innocent and suspect groups.

The analysis of Figure 9 leads to the following conclusion. When distinguishing between the innocent and suspect groups, an accuracy of 85% can be achieved using only three features and 87% using eight features. Although the model achieved a maximum accuracy of 88.4%, the numbers of features used at this point were 22, 28, and 30, and the Kappa coefficient at this point was 0.82, which has a fairly satisfactory degree of agreement (Kappa ≥ 0.8 is generally considered to be almost perfect agreement). The first three features were the fixation time, which indicates that the gaze duration can reflect the familiarity of the participants with the area of interest and the processing load. The accuracy of the model decreased when the fourth feature $GR.\left(T^{\prime }P_{2}I_{f}\right)$ was added. From the data, it can be seen that there is no fixed pattern in the fixation count the three groups gazed at the non-involved items, which led to the decrease in the model accuracy.

**Figure 10:**
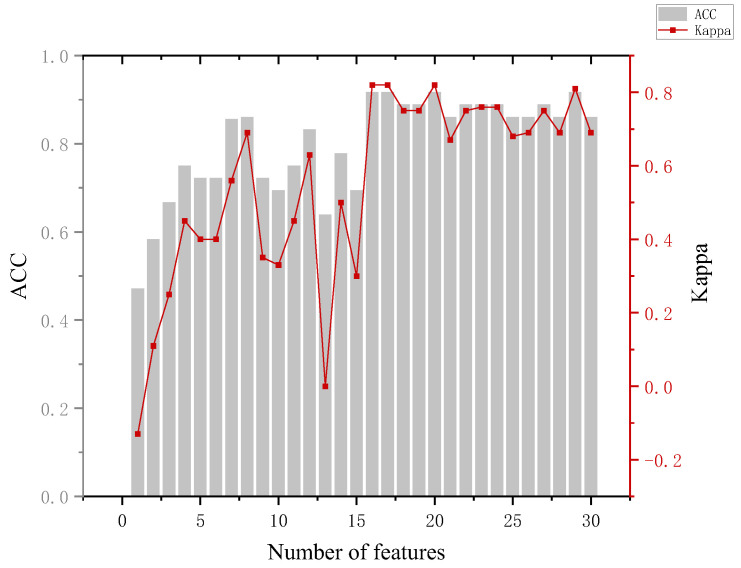
Results for the innocent and crime groups.

The analysis of Figure 10 leads to the following conclusion. The highest accuracy of 91.7% of the model in distinguishing between the innocent and perpetrator group results occurred when 16, 17, 20, and 29 features were used, but the accuracy of the model could reach 86.1% when 8 features were used and the Kappa coefficient was 0.69, and we considered that the effect of classification was highly consistent at this time. (It is generally considered to be highly consistent when 0.6 < Kappa < 0.8).

**Figure 11:**
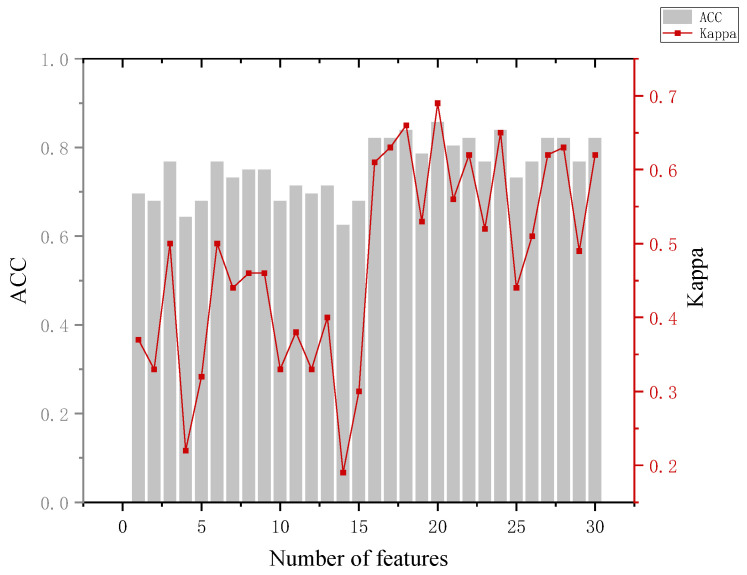
Results of the informed and crime groups.

The analysis of Figure 11 leads to the following conclusion. In distinguishing between the informed and perpetrator groups, the highest accuracy of the model of 85.7% occurred, which required 20 features and did not show better classification when using a smaller number of features. Looking at the mean of and variance in the eye-movement data between the informed and perpetrator groups also revealed that the difference was small, and even the informed group showed a greater overall stimulation than the perpetrator group in the identification of some key crime episodes. The reason for this may be that the stimuli were perceived in accordance with the expectation rather than the actual physical stimuli during the recall process, and the phenomenon of perceptual fixation occurred.

**Figure 12:**
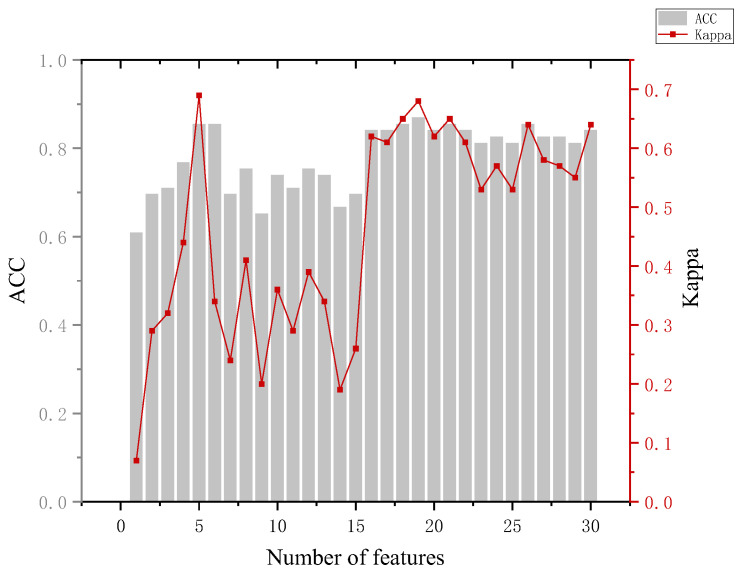
Results for the crime and non-crime groups.

The analysis of Figure 12 leads to the following conclusion. The highest accuracy of the model of 87% occurred when distinguishing between the crime and non-crime groups. With the addition of the first five features in turn, the model effect gradually improved and could reach an accuracy of 85.5%, and the Kappa coefficient was 0.69 at this time. The difference was whether the informed group was included in the classification compared to the innocent and crime groups. The results show that when considering the informed group, although the highest accuracy of the model is reduced, the model can clearly use a smaller number of features to make the identity judgments of the crime and non-crime groups.

The analysis of the ranking of the importance of features shows that features in the category of fixation time and fixation count have better results for identification. This can also be further explained according to the eye-brain hypothesis [35], responding to the processing of that process by the participants; also, We can observe that increasing the number of features does not necessarily result in higher accuracy in model classification.

## 5. Conclusions

By constructing the RF-RFE model, we can not only obtain the importance ranking of features, but also use the ranking to seek the best model for identification in order to achieve a higher accuracy rate using a smaller number of features.

The results showed that (1) under the simulated crime scenario, in the innocent group and the informed group, when faced with the same pressure, significant differences were observed among different groups in indicators such as scene fixation time, scene fixation count, and portrait pupil diameter. (2) Based on the interpretable machine learning approach, it can be found that the five features, namely, the length of involved object gaze, the length of involved portrait gaze, the length of uninvolved scene fixation time, the number of uninvolved object fixation time, and the length of uninvolved object fixation time, contribute the most to the accuracy of the model’s prediction. The model accuracy can reach 84.8–91.7% for different classification cases. The attentional category indicators can better reflect the differences in participants’ familiarity and processing levels regarding the circumstances involved in the case and can achieve effective identity differentiation.

As for the section on future work, we have also given it careful consideration. This paper primarily focuses on fundamental research. Currently, there is a lack of eye-movement data from individuals involved in real criminal cases for analysis and reference. Conducting research under controlled laboratory conditions makes it challenging to simulate the psychological state following an actual crime, and the effectiveness of this method in practical tests within the field of criminal investigation remains unverified. Furthermore, due to the impact of COVID-19, the experiment could only be conducted within the school premises. Additionally, the participant selection process was limited and the number of participants varied slightly across groups. Therefore, in future work, we can explore the feasibility of using eye-movement indicators to construct identity recognition models by designing more realistic simulated crime scenarios or applying them in actual identification processes. Expanding the range of participant selection would allow for further investigation into the effectiveness of these eye-movement indicators in the identification process.

## Figures and Tables

**Table 1:** RF-RFE feature ranking results.

Sequence	Variable	Sequence	Variable	Sequence	Variable
1	$GR.\left(TP_{2}I_{f}\right)$	11	$GR.\left(T^{\prime }P_{3}I_{c}\right)$	21	$GR.\left(T^{\prime }P_{1}I_{f}\right)$
2	$GR.\left(TP_{1}I_{f}\right)$	12	$GR.\left(T^{\prime }P_{1}I_{b}\right)$	22	$GR.\left(TP_{1}I_{s}\right)$
3	$GR.\left(T^{\prime }P_{3}I_{f}\right)$	13	$GR.\left(TP_{1}I_{b}\right)$	23	$GR.\left(TP_{2}I_{b}\right)$
4	$GR.\left(T^{\prime }P_{2}I_{c}\right)$	14	$GR.\left(T^{\prime }P_{1}I_{d}\right)$	24	$GR.\left(TP_{3}I_{b}\right)$
5	$GR.\left(T^{\prime }P_{2}I_{f}\right)$	15	$GR.\left(TP_{1}I_{c}\right)$	25	$GR.\left(T^{\prime }P_{3}I_{d}\right)$
6	$GR.\left(T^{\prime }P_{1}I_{c}\right)$	16	$GR.\left(TP_{3}I_{c}\right)$	26	$GR.\left(TP_{3}I_{d}\right)$
7	$GR.\left(T^{\prime }P_{2}I_{b}\right)$	17	$GR.\left(TP_{3}I_{s}\right)$	27	$GR.\left(TP_{1}I_{d}\right)$
8	$GR.\left(T^{\prime }P_{2}I_{d}\right)$	18	$GR.\left(T^{\prime }P_{1}I_{s}\right)$	28	$GR.\left(TP_{2}I_{d}\right)$
9	$GR.\left(T^{\prime }P_{3}I_{b}\right)$	19	$GR.\left(T^{\prime }P_{3}I_{s}\right)$	29	$GR.\left(TP_{2}I_{s}\right)$
10	$GR.\left(TP_{2}I_{c}\right)$	20	$GR.\left(T^{\prime }P_{2}I_{s}\right)$	30	$GR.\left(TP_{3}I_{f}\right)$

## Data Availability

Due to the involvement of human participants in the experimental data, the relevant data is treated as confidential.

## Appendix A

**Table A1:** Means and standard deviations of eye-movement indicators under portrait stimulation.

		Portrait				
Category	AOI	Fixation Time	Fixation Count	Pupil Diameter	Saccade Frequency	Blink Frequency
Innocent	Involved	1201.64 ± 916.05	3.77 ± 2.68	3.98 ± 0.59	2.98 ± 0.77	0.59 ± 0.29
	Uninvolved	1128.47 ± 740.79	4.05 ± 2.71	3.91 ± 0.53	3.01 ± 0.34	0.59 ± 0.22
Informed	Involved	1328.44 ± 881.53	5.03 ± 3.27	4.31 ± 0.45	3.16 ± 0.79	0.43 ± 0.37
	Uninvolved	1075.04 ± 769.88	4.21 ± 2.86	4.06 ± 0.42	3.07 ± 0.68	0.47 ± 0.30
Crime	Involved	2117.03 ± 956.41	7.00 ± 2.88	4.73 ± 0.47	2.90 ± 0.94	0.36 ± 0.30
	Uninvolved	1231.25 ± 877.16	4.70 ± 3.16	4.28 ± 0.38	3.00 ± 0.69	0.35 ± 0.21

**Table A2:** Means and standard deviations of eye-movement indicators under object stimulation.

		Object				
Category	AOI	Fixation Time	Fixation Count	Pupil Diameter	Saccade Frequency	Blink Frequency
Innocent	Involved	1183.18 ± 648.78	4.15 ± 2.44	3.86 ± 0.50	4.42 ± 0.31	0.35 ± 0.34
	Uninvolved	824.53 ± 336.71	3.44 ± 1.24	3.90 ± 0.49	3.38 ± 0.32	0.45 ± 0.18
Informed	Involved	1586.08 ± 840.08	6.46 ± 3.22	4.36 ± 0.48	3.42 ± 0.92	0.25 ± 0.28
	Uninvolved	624.63 ± 280.03	2.79 ± 1.20	4.50 ± 2.13	3.32 ± 0.64	0.34 ± 0.25
Crime	Involved	1214.04 ± 679.00	5.39 ± 2.92	4.54 ± 0.42	3.31 ± 0.96	0.30 ± 0.27
	Uninvolved	646.20 ± 274.59	2.97 ± 1.15	4.42 ± 0.31	3.39 ± 0.70	0.27 ± 0.19

**Table A3:** Means and standard deviations of eye-movement indicators under scene stimulation.

		Scene				
Category	AOI	Fixation Time	Fixation Count	Pupil Diameter	Saccade Frequency	Blink Frequency
Innocent	Involved	368.03 ± 363.73	1.54 ± 1.16	4.03 ± 0.60	3.52 ± 0.66	0.46 ± 0.24
	Uninvolved	426.12 ± 150.17	1.70 ± 0.59	3.90 ± 0.47	3.36 ± 0.50	0.49 ± 0.23
Informed	Involved	754.68 ± 328.78	2.92 ± 1.10	4.49 ± 0.55	3.46 ± 0.79	0.33 ± 0.28
	Uninvolved	256.37 ± 112.58	1.15 ± 0.49	4.21 ± 0.43	3.42 ± 0.77	0.33 ± 0.24
Crime	Involved	1363.66 ± 394.62	5.54 ± 1.64	4.86 ± 0.42	3.42 ± 0.60	0.30 ± 0.21
	Uninvolved	289.74 ± 141.96	1.30 ± 0.52	4.35 ± 0.25	3.39 ± 0.54	0.32 ± 0.23

**Table A4:** Results of RF-RFE model: (**a**) Innocent and informed groups; (**b**) innocence and suspicion (informed and crime) groups; (**c**) innocence and crime groups; (**d**) informed and crime groups; and (**e**) crime and non-crime groups.

(a)								
Number	ACC	Kappa	Number	ACC	Kappa	Number	ACC	Kappa
1	63.0%	0.11	11	73.9%	0.33	21	76.1%	0.37
2	52.2%	−0.18	12	73.9%	0.33	22	80.4%	0.45
3	73.9%	0.36	13	84.8%	0.60	23	71.7%	0.00
4	73.9%	0.33	14	69.6%	0.28	24	73.9%	0.25
5	71.7%	0.21	15	71.7%	0.25	25	80.4%	0.42
6	67.4%	0.18	16	73.9%	0.25	26	87.0%	0.63
7	71.7%	0.25	17	78.3%	0.38	27	80.4%	0.45
8	73.9%	0.29	18	76.1%	0.25	28	78.3%	0.38
9	71.7%	0.25	19	82.6%	0.47	29	84.8%	0.60
10	69.6%	0.08	20	84.8%	0.60	30	80.4%	0.48
(**b**)								
**Number**	**ACC**	**Kappa**	**Number**	**ACC**	**Kappa**	**Number**	**ACC**	**Kappa**
1	66.7%	0.60	11	84.1%	0.81	21	85.5%	0.84
2	65.2%	0.58	12	79.7%	0.75	22	88.4%	0.84
3	85.5%	0.82	13	81.2%	0.77	23	82.6%	0.84
4	78.3%	0.73	14	84.1%	0.80	24	85.5%	0.84
5	75.4%	0.70	15	84.1%	0.80	25	84.1%	0.84
6	84.1%	0.80	16	85.5%	0.82	26	87.0%	0.84
7	75.4%	0.70	17	84.1%	0.80	27	87.0%	0.84
8	87.0%	0.84	18	81.2%	0.77	28	88.4%	0.84
9	79.7%	0.75	19	81.2%	0.84	29	84.1%	0.84
10	82.6%	0.79	20	82.6%	0.84	30	88.4%	0.84
(**c**)								
**Number**	**ACC**	**Kappa**	**Number**	**ACC**	**Kappa**	**Number**	**ACC**	**Kappa**
1	47.2%	−0.13	11	75.0%	0.45	21	86.1%	0.67
2	58.3%	0.11	12	83.3%	0.63	22	88.9%	0.75
3	66.7%	0.25	13	63.9%	0.00	23	88.9%	0.76
4	75.0%	0.45	14	77.8%	0.50	24	88.9%	0.76
5	72.2%	0.40	15	69.4%	0.30	25	86.1%	0.68
6	72.2%	0.40	16	91.7%	0.82	26	86.1%	0.69
7	85.6%	0.56	17	91.7%	0.82	27	88.9%	0.75
8	86.1%	0.69	18	88.9%	0.75	28	86.1%	0.69
9	72.2%	0.35	19	88.9%	0.75	29	91.7%	0.81
10	69.4%	0.33	20	91.7%	0.82	30	86.1%	0.69
(**d**)								
**Number**	**ACC**	**Kappa**	**Number**	**ACC**	**Kappa**	**Number**	**ACC**	**Kappa**
1	69.6%	0.37	11	71.4%	0.38	21	80.4%	0.56
2	67.9%	0.33	12	69.6%	0.33	22	82.1%	0.62
3	76.8%	0.50	13	71.4%	0.40	23	76.8%	0.52
4	64.3%	0.22	14	62.5%	0.19	24	83.9%	0.65
5	67.9%	0.32	15	67.9%	0.30	25	73.2%	0.44
6	76.8%	0.50	16	82.1%	0.61	26	76.8%	0.51
7	73.2%	0.44	17	82.1%	0.63	27	82.1%	0.62
8	75.0%	0.46	18	83.9%	0.66	28	82.1%	0.63
9	75.0%	0.46	19	78.6%	0.53	29	76.8%	0.49
10	67.9%	0.33	20	85.7%	0.69	30	82.1%	0.62
(**e**)								
**Number**	**ACC**	**Kappa**	**Number**	**ACC**	**Kappa**	**Number**	**ACC**	**Kappa**
1	60.9%	0.07	11	71.0%	0.29	21	85.5%	0.65
2	69.6%	0.29	12	75.4%	0.39	22	84.1%	0.61
3	71.0%	0.32	13	73.9%	0.34	23	81.2%	0.53
4	76.8%	0.44	14	66.7%	0.19	24	82.6%	0.57
5	85.5%	0.69	15	69.6%	0.26	25	81.2%	0.53
6	85.5%	0.34	16	84.1%	0.62	26	85.5%	0.64
7	69.6%	0.24	17	84.1%	0.61	27	82.6%	0.58
8	75.4%	0.41	18	85.5%	0.65	28	82.6%	0.57
9	65.2%	0.20	19	87.0%	0.68	29	81.2%	0.55
10	73.9%	0.36	20	84.1%	0.62	30	84.1%	0.64
